# Association between periodontal condition and kidney dysfunction in Japanese adults: A cross‐sectional study

**DOI:** 10.1002/cre2.39

**Published:** 2016-08-11

**Authors:** Koji Naruishi, Keiji Oishi, Yuji Inagaki, Masumi Horibe, Mika Bando, Masami Ninomiya, Kazuhiko Kawahara, Jun Minakuchi, Shu Kawashima, Kenji Shima, Jun‐ichi Kido, Toshihiko Nagata

**Affiliations:** ^1^ Department of Periodontology and Endodontology, Institute of Biomedical Sciences Tokushima University Graduate School Tokushima Tokushima Prefecture Japan; ^2^ Kawashima Hospital Kitasako Ichibancho Tokushima Tokushima Prefecture Japan

**Keywords:** diabetes mellitus, estimated glomerular filtration rate, kidney dysfunction, periodontitis

## Abstract

Recent studies have demonstrated that chronic kidney disease (CKD) may be associated with the progression of periodontal disease. Diabetes mellitus (DM) is a major risk factor for CKD. The objective of this study was to clarify the relationship between periodontal condition and kidney dysfunction in patients who had kidney failure with or without DM. One hundred sixty‐four patients with kidney dysfunction were enrolled (male: *N* = 105; female: *N* = 59), and the relationship between periodontal condition and kidney dysfunction was analyzed in a cross‐sectional study. The subjects were divided into three groups: (a) patients with DM, (b) dialysis patients with nephropathy due to various kidney diseases, and (c) dialysis patient with nephropathy due to DM (diabetic nephropathy). Then, the effect of DM on the periodontal condition was analyzed. The patients were also stratified by CKD stage (into G1–G5) using the estimated glomerular filtration rate (eGFR), and the G5 group was divided in patients with or without DM. Correlations between eGFR and parameters of periodontal condition were calculated in patients from G1 to G4. The number of missing teeth was significantly higher in dialysis patients with diabetic nephropathy than in patients with DM, whereas alveolar bone loss did not show a significant difference among the three groups. In addition, the G5 patients with DM had a significantly higher number of missing teeth than the other CKD groups, whereas alveolar bone loss did not show a significant difference. In G5 patients with DM, Community Periodontal Index and Oral Hygiene Index scores were significantly higher than in G1‐4 patients with DM. There was a significant negative correlation between eGFR and the number of missing teeth. Patients with diabetic nephropathy have a higher rate of periodontal problems such as missing teeth in Japanese adults.

## 1 INTRODUCTION

The number of patients with lifestyle diseases such as diabetes mellitus (DM) has been steadily increasing worldwide. DM is an important health problem because it has become the leading cause of chronic kidney disease (CKD; Nashar & Egan, 2014), and the concomitant conditions might affect the quality of life of patients. It has been reported that diabetic nephropathy occurs in up to 40% of patients with type 1 or type 2 DM (Macisaac, Ekinci, & Jerums, 2014). Diabetic nephropathy is a significant risk factor of progression to end‐stage renal disease, resulting in the need for dialysis. In Japan, it is estimated that >314,000 patients were on maintenance dialysis for end‐stage renal disease in 2013 (Watanabe et al., 2015).

Periodontitis is a multifactorial chronic inflammatory disease that causes tooth loss. It is characterized by destruction of connective tissue, the periodontal ligament, and alveolar bone, leading to a significant increase of systemic exposure to bacteria (Okada et al., 1996). Many investigators have suggested a two‐way relationship between diabetes and periodontitis (Nagasawa et al., 2010; Nishimura et al., 2003; Preshaw et al., 2012). Interestingly, it has been reported that periodontal infection shows a positive correlation with endothelial dysfunction (Tonetti et al., 2007) and that infection with *Porphyromonas gingivalis* increases endothelial injury in obese mice (Ao et al., 2014). Furthermore, intensive periodontal treatment improves endothelial function (Tonetti et al., 2007). It is possible that circulating periodontal bacteria could damage the renal endothelium (Tonetti et al., 2007). Because alteration of endothelial function could lead to reduction of renal blood flow, periodontal infection might consequently worsen kidney function (Tonetti et al., 2007). A recent cohort study showed that severe periodontitis is associated with kidney dysfunction in African Americans (Grubbs et al., 2015). Other clinical studies have also demonstrated a close association between CKD and periodontal disease (Ioannidou & Swede, 2011; Iwasaki et al., 2012).

Although the glomerular filtration rate is a useful index for the assessment of CKD, it cannot be measured directly in daily clinical practice (Wyatt, Konduri, Eng, & Rohatgi, 2007). Clinically, the most common useful marker for renal disease and progression of CKD might well be the estimated glomerular filtration rate (eGFR). Kidney function is often classified into G1, G2, G3, G4, and G5 stages based on eGFR values to select treatment (Rosansky, 2012). Although the timing for initiation of dialysis remains controversial, many physicians recommend initiating the dialysis therapy when the eGFR is <15 ml min^−1^ 1.73 m^−^^2^ (Churchill, 1997), so this recommendation has an impact on both clinical practice and medical expenses. It has been found that periodontal treatment improves the eGFR in CKD patients (Chambrone et al., 2013).

Our hypothesis is that progression of periodontitis might be more rapid when kidney disease is more advanced, such as in dialysis patients. In the present study, therefore, we investigated whether periodontal condition was correlated with the severity of kidney dysfunction. The objective of this cross‐sectional study was to assess the relation between periodontal condition and the stage of kidney dysfunction in patients with or without DM.

## 2 MATERIALS AND METHODS

### 2.1 Subjects and evaluations

One hundred sixty‐four patients with kidney dysfunction who regularly visited the outpatient clinic of Kawashima Hospital (Tokushima, Japan) were enrolled in this study (male: 60.1 ± 10.5 years, *N* = 105; female: 62.3 ± 11.1 years, *N* = 59). We determined the age, sex, smoking history including both past and present history, systolic blood pressure (BP), and body mass index (BMI) from the medical records of eligible patients. We also examined laboratory data, including the postprandial blood sugar level (BS), hemoglobin A1c (HbA1c), blood urea nitrogen, serum uric acid, serum creatinine (Cr), serum Ca/P, serum β2‐microglobulin, and hematocrit at the first visit. DM was diagnosed by a specialist physician. Initiation of dialysis treatment was decided by clinical assessment. The formula proposed by the Japanese Society of Nephrology in 1997 was applied for calculation of eGFR as follows:

eGFR (ml min^−1^ 1.73 m^2^) = 194 × serum Cr^−^^1.094^ × age^−^^0.287^ (×0.739 in women).

In the Conference Report, CKD is characterized by both the eGFR and the stage of albuminuria (Levey, Inker, & Coresh, 2014), although eGFR is considered to be the best measure of kidney function and best determinant of the stage of CKD, as reported by the National Kidney Foundation in 2002. After enrollment in this study, periodontal evaluation was performed promptly by trained dentists. The Community Periodontal Index (CPI) recommended by the World Health Organization was determined to assess periodontitis (Ainomo et al., 1982). Oral hygiene was assessed by using the Oral Hygiene Index (OHI) Simplified scale (Greene & Vermillion, 1964). The number of missing teeth was counted and recorded by trained dentists. Assessment of alveolar bone loss was performed by calculating bone loss as a proportion of the tooth root length on X‐ray films using a Schei ruler (Bassiouny & Grant, 1975). Trained dentists performed all periodontal examination, and a supervisory dentist checked it so that there was no difference in technique among attending dentists.

Informed consent was obtained from each patient prior to enrollment in this study, which was performed in accordance with the Declaration of Helsinki and was approved by the Ethics Committee of Kawashima Hospital.

### 2.2 Statistical analysis

Numerical variables are presented as the mean and standard deviation, while categorical variables were presented as frequencies and percentages. First, the periodontal condition was compared statistically among patients with DM, hemodialysis patients, and hemodialysis patients with DM. Next, the periodontal condition was compared between patients in renal function groups G1–G4 and patients in G5. To analyze the effect of DM in more depth, the G5 group was divided into patients with or without DM. Finally, the correlation between eGFR and periodontal statues were calculated in patients with stage of G1–G4. Statistical differences were compared by using the chi‐square test, analysis of variance (ANOVA), or Spearman's rank correlation coefficient, as appropriate, and the differences between groups were assessed with the Tukey–Kramer honestly significant difference (HSD) test. The odds ratios derived from bivariate analysis with regard to gender and 95% confidence interval were obtained by conditional logistic regression analysis. Statistical analyses were performed using JMP® 8 ver. 8.0.2 (SAS Institute Japan, Tokyo). Probability (*p*) values of less than .05 were considered statistically significant.

## 3 RESULTS

### 3.1 Basic characterization and periodontal condition of each group

The subjects were categorized into a DM group (M/F: 28/20), a hemodialysis group (M/F: 51/33), and a hemodialysis with DM group (M/F: 26/6), and their characteristics are shown in Table 1. There was a significant difference in the ratio of men between the hemodialysis and hemodialysis with DM groups (odds ratio: 2.80; 95% confidence interval [1.1, 7.3]; *p* = .036). There were no significant differences among the groups in terms of age, BMI, and smoking, although the smoking rate of the hemodialysis with DM group was slightly higher than those of the other groups (vs. DM group, *p* = .14; vs. hemodialysis group, *p* = .16, chi‐square test). In addition, there was a significant difference of BP between the DM and hemodialysis with DM groups (*p* = .0010, ANOVA with Tukey HSD). DM‐related laboratory data tended to be lower in the hemodialysis with DM group compared with the DM group, although the differences were not significant (BS: *p* = .44, HbA1c: *p* = .34, ANOVA with Tukey HSD).

**Table 1:**
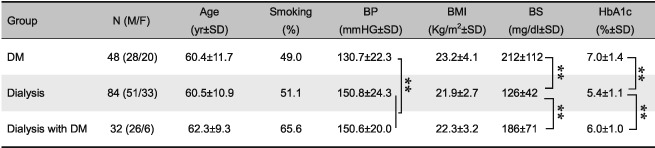
Basic characterization of each group

*Note*: Period of dialysis: dialysis group, 13.7 ± 22.7 years; dialysis with DM group, 4.4 ± 5.0 years. ANOVA = analysis of variance; BP = systolic blood pressure; BMI = body mass index; BS = postprandial blood sugar level; HbA1c = hemoglobin A1c; DM = diabetes mellitus; F = female; M = male; HSD, honestly significant difference; SD, standard deviation

** *p* < .01, ANOVA with Tukey HSD test

**Figure 1:**
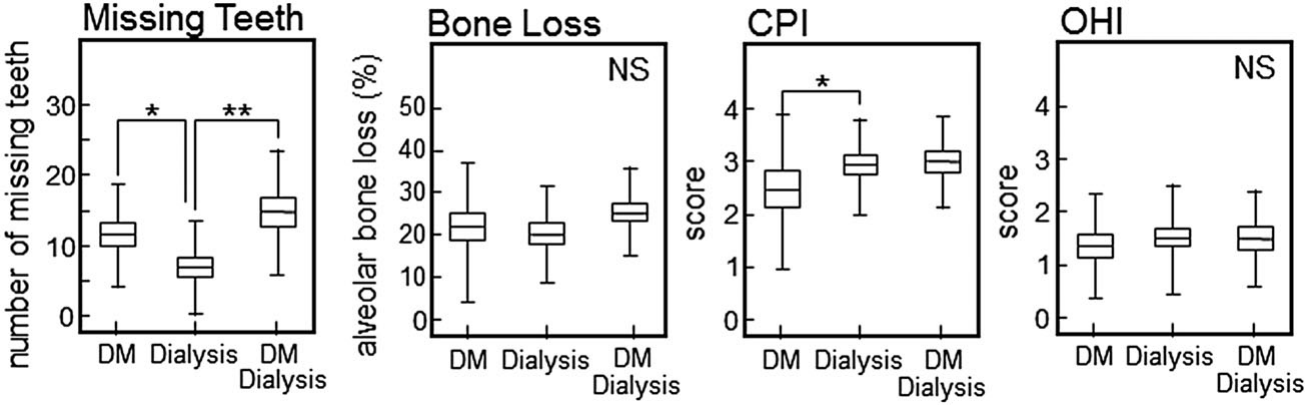
Comparison of the periodontal condition in each group. We examined the following periodontal parameters: missing teeth, bone loss, CPI, and OHI. Data are expressed as the mean ± SD. CPI scores of 0–4 were assigned: *healthy and absence of gingival bleeding* (score 0), *gingival bleeding* (score 1), *presence of supragingival or subgingival calculus* (score 2), *probing pocket depth of 4–5 mm* (score 3), and *probing pocket ≥6 mm* (score 4). The OHI is composed of the plaque index and calculus index, with each of these indices being based on 12 numerical determinations representing the amount of plaque or calculus on the buccal and lingual surfaces of each of three segments of each dental arch. *p < .05, **p < .01, ANOVA with Tukey HSD. NS = not significant. DM = diabetes mellitus; SD = standard deviation; CPI, WHO Community Periodontal Index; OHI, Oral Hygiene Index; ANOVA = analysis of variance; HSD, honestly significant difference

Next, Figure 1 shows differences of the periodontal condition among the groups. The number of missing teeth was significantly higher in the hemodialysis with DM group than in the hemodialysis group (*p* = .0001, ANOVA with Tukey HSD). In addition, the number of missing teeth was slightly, but not significantly, higher in the hemodialysis with DM group than in the DM group (*p* = .097, ANOVA with Tukey HSD). Although there were no significant differences among the groups with regard to alveolar bone loss, it tended to be higher in the hemodialysis with DM group compared with the hemodialysis group (*p* = .079, ANOVA with Tukey HSD). The CPI score was lower in the DM group than in the other group (*p* = .083, vs. hemodialysis with DM; *p* = .033, vs. hemodialysis, ANOVA with Tukey HSD). There were no significant differences of the OHI score among the groups (*p* = .84, hemodialysis vs. hemodialysis with DM, ANOVA with Tukey HSD).

### 3.2 Comparison of each group categorized by CKD stage

**Figure 2:**
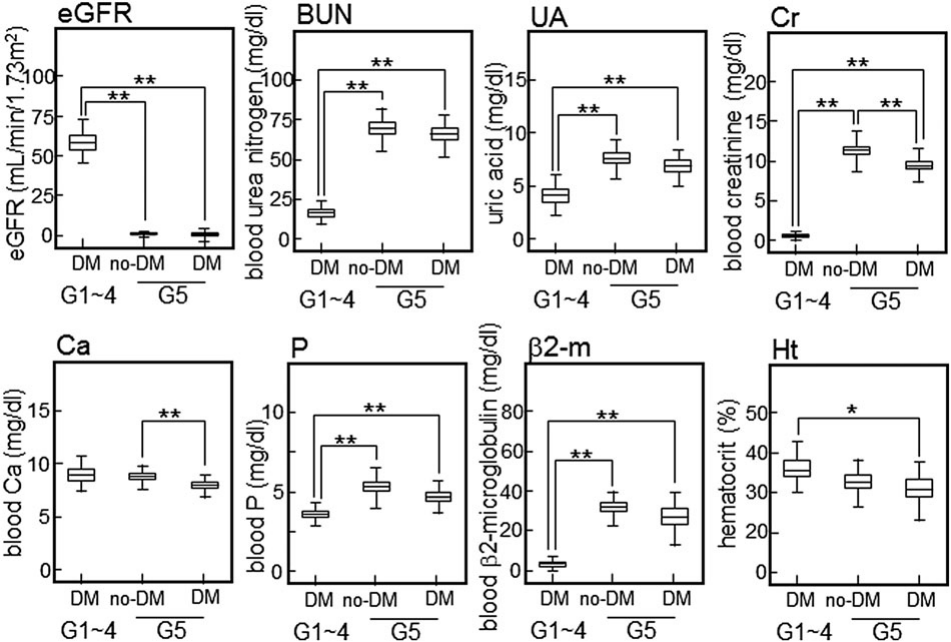
Kidney function in each group. Laboratory tests (eGFR, UA, Cr, Ca/P, β2‐m, and Ht) were performed routinely to evaluate kidney function. Sample size: G1–4 with DM, *N* = 45; G5 without DM, *N* = 84; G5 with DM, *N* = 35. *p < .05, **p < .01, ANOVA with Tukey HSD. DM = diabetes mellitus; ANOVA = analysis of variance; HSD, honestly significant difference; eGFR = estimated glomerular filtration rate; BUN = blood urea nitrogen; UA = serum uric acid; Cr = serum creatinine; β2‐m = serum β2‐microglobulin; Ht, hematocrit

As shown in Table 2, we compared the characteristics of each group categorized by CKD stage (divided into G1, G2, G3, G4, and G5 based on eGFR). There were no significant differences among each CKD stage with respect to age, BMI, and postprandial BS level, although eGFR decreases with aging (Liljestrand et al., 2015). BP was significantly higher in the G5 group than in the G2 or G3 groups (G2: *p* = .0010, G3: *p* = .0044, ANOVA with Tukey HSD). The HbA1c values were significantly lower in the G5 group than in the G2 or G3 groups (G2: *p* = 0.0002, G3: *p* = 0.0009, ANOVA with Tukey HSD). Next, the subjects with stages 1, 2, 3, and 4 CKD were combined into one group for analysis because of small sample sizes. As shown in the lower part of Table 2, almost all of the subjects in G5 group were on hemodialysis therapy, while almost all subjects in the G1‐4 group were not on hemodialysis therapy. The G5 group was further divided into two groups with or without DM. There were no significant differences of age and BMI between these groups. In each G5 subgroup, BP was significantly higher than in the G1‐4 with DM group. As expected, DM‐related laboratory data were significantly lower in the G5 without DM group than in both the G1‐4 group and the G5 with DM group. In addition, we examined the validity of our classification based on laboratory tests for evaluation of kidney function. As shown in Figure 2, the eGFR was significantly higher in the G1‐4 with DM group than in both G5 subgroups. The levels of blood urea nitrogen, serum uric acid, Cr, P, and β2‐microglobulin were significantly lower in the G1‐4 with DM group than in both G5 subgroups. Interestingly, we found that there were significant differences of blood Cr and Ca levels between the G5 with DM group and the G5 without DM group (Cr, *p* = .0002; Ca, *p* = .0010, ANOVA with Tukey HSD). There was significant difference of the hematocrit level between the G1‐4 group and the G5 with DM group (*p* = .046, ANOVA with Tukey HSD).

**Table 2:**
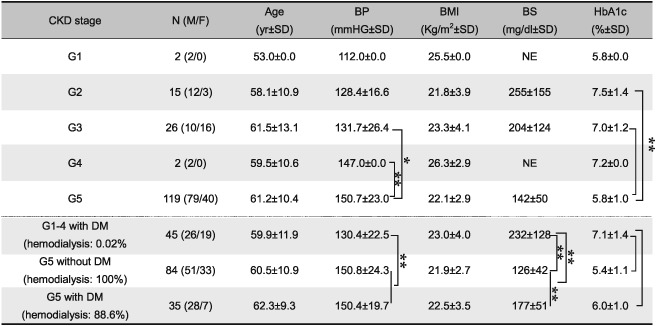
Comparison of each group categorized by CKD stage

*Note*: Stages of CKD were categorized by eGFR values in Japanese Society of Nephrology on 2012 (G1, ≥90; G2, 60–89; G3, 30–59, G4, 15–29; and G5, <15 ml min^−1^ 1.73 m^−2^). Due to the small sample sizes, subjects with CKD stage 1, 2, 3, and 4 were combined into one group for comparative analyses. All of subjects in G1–4 group had contracted DM. CKD = chronic kidney disease; BP = systolic blood pressure; BMI = body mass index; BS = postprandial blood sugar level = HbA1c, hemoglobin A1c; DM = diabetes mellitus. NE = not examined; F = female; M = male; SD, standard deviation; eGFR = estimated glomerular filtration rate; ANOVA = analysis of variance; HSD, honestly significant difference

* *p* < .05

** *p* < .01, ANOVA with Tukey HSD test

**Figure 3:**
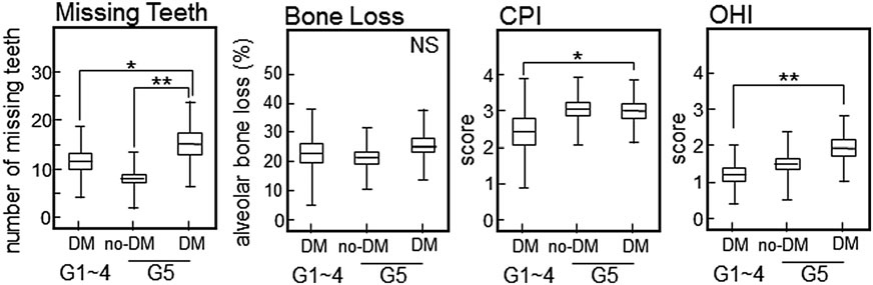
Comparison of the periodontal condition among each group categorized by CKD stage (into G1–G5). Periodontal conditions were evaluated just like in Figure 1. *p < .05, **p < .01, ANOVA with Tukey HSD. NS = not significant; CPI = WHO Community Periodontal Index; OHI = Oral Hygiene Index; DM = diabetes mellitus; ANOVA = analysis of variance; HSD, honestly significant difference

Figure 3 shows differences of the periodontal condition among each group categorized by CKD stage. The number of missing teeth was significantly higher in the G5 with DM group than in the other groups (*p* = .045, vs. G1‐4 with DM; *p* < .0001, vs. G5 without DM, ANOVA with Tukey HSD). Although there were no significant differences among each group with regard to alveolar bone loss, it tended to be higher in the G5 with DM group compared with the G5 without DM group (*p* = .071, ANOVA with Tukey HSD). CPI and OHI scores were significantly higher in the G5 with DM group than in the G1‐4 with DM group (CPI, *p* = .026: OHI, *p* = .026, ANOVA with Tukey HSD).

### 3.3 Correlation between eGFR and several parameters

**Figure 4:**
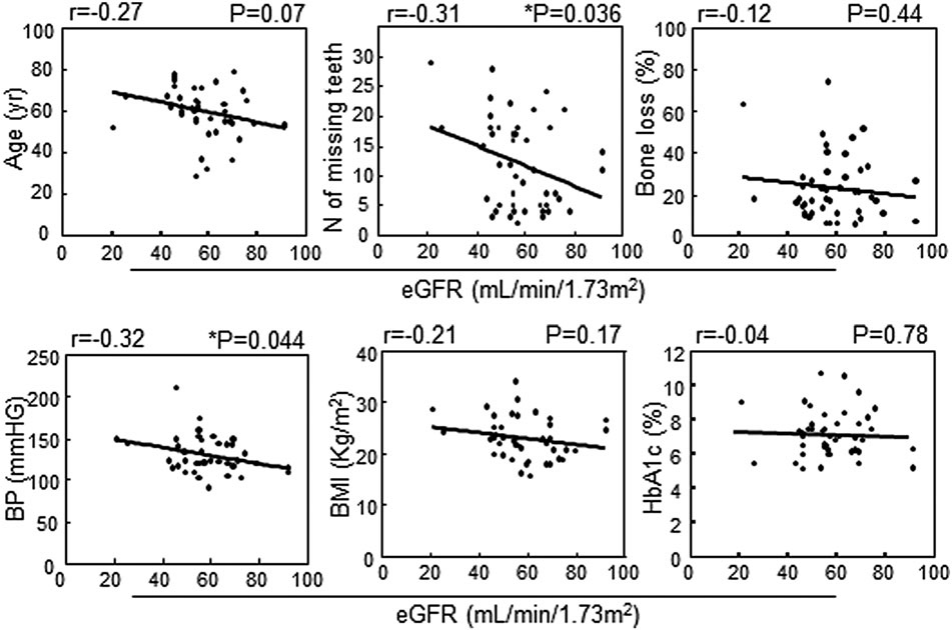
Correlations between eGFR and several parameters in the G1–4 group. Spearman's rank correlation analysis method was used for statistical analysis and *p*‐values of less than .05 were considered statistically significant. eGFR, estimated glomerular filtration rate; BP = systolic blood pressure

Correlations were examined between eGFR and several parameters. As shown in Figure 4, there was a significant negative correlation between eGFR and BP (*p* = .044, *r* = −.32, Spearman's rank correlation test). In the case of periodontal condition parameters, we found that the number of missing teeth, but not bone loss, also showed a significant negative correlation with eGFR (*p* = .036, *r* = −.31, Spearman's rank correlation test). On the other hand, there was no significant correlation between eGFR and age, BMI, or HbA1c.

## 4 DISCUSSION

The kidneys are a vital excretory organ, and renal disease is a major health problem worldwide (Williams, 2015). Unfortunately, many patients with DM develop kidney dysfunction, and the high prevalence of microvascular complications caused by hyperglycemia may be a major reason for the onset of renal disease. CKD is defined as either kidney dysfunction or an eGFR <60 ml min^−1^ m^−2^ persisting for more than 3 months according to the National Kidney Foundation on 2002, and progression of CKD eventually leads to the need for hemodialysis or kidney transplantation due to kidney failure. Hemodialysis is a long‐term therapy that is performed once, twice, or more often per week. Therefore, hemodialysis increases the stress on patients, although it reduces morbidity and mortality among patients with end‐stage renal disease.

It has been generally accepted that periodontitis could be an important risk factor for CKD and kidney failure (Chambrone et al., 2013) based on the following two points: (a) systemic inflammation is affected by periodontal inflammation and (b) periodontal bacteria and/or their products can enter the bloodstream (Kshirsagar et al., 2005). In addition, the oral health of patients undergoing dialysis therapy is frequently found to be poor due to insufficient oral hygiene caused by their poor condition. Longitudinal studies have demonstrated a two‐way relationship between DM and periodontitis, that is, progression of periodontitis occurs in patients with DM and deterioration of glycemic control is induced by periodontal inflammation in DM patients (Llambés, Arias‐Herrera, & Caffesse, 2015). In the present study, we statistically evaluated the relation between periodontal health and kidney dysfunction in patients with or without DM.

Although the incidence of DM is not different between men and women in general, specific male responses such as testosterone regulation may influence the development of diabetic complications (Bao & Johansen, 2015). However, further investigation will be needed in the future to clarify the factors involved. In addition to DM, hypertension and smoking contribute to endothelial and vascular smooth muscle cell damage, resulting in progression of renal disease and cardiovascular sequelae.

Because advanced tooth loss reflects the cumulative effects of past of oral inflammation such as periodontitis, it has been suggested that missing teeth may be a factor in determining the risk of systemic diseases (Figure 1). Recently, it was reported that the number of missing teeth may indicate an increased risk of cardiovascular disease, DM, or all‐cause mortality (Liljestrand et al., 2015). Furthermore, Ioannidou et al. has reported that poor oral health as manifested by tooth loss predicts malnutrition in CKD patients of the United States population (Ioannidou, Swede, Fares, & Himmelfarb, 2014). We also showed that the number of missing teeth increased significantly in DM patients with kidney failure (Figure 1A). Although there were no significant differences among each group in relation to bone loss, we considered that the finding is reasonable because bone loss did not reflect the number of missing teeth. However, bone loss was slightly higher in the hemodialysis with DM group than in the hemodialysis without DM group (hemodialysis with DM: 27.0% ± 8.8%, hemodialysis without DM: 20.7% ± 10.6%, *p* = .46). In addition, a representative index of periodontitis (the CPI score) was higher in both hemodialysis groups than in the DM group (hemodialysis: *p* = .033, hemodialysis with DM: *p* = .083). We consider that periodontitis is highly prevalent in chronic renal failure patients who have unacceptably poor oral hygiene, suggesting that oral health promotion and prevention programs are needed for patients on hemodialysis.

Because eGFR was less than 15 ml min^−1^ 1.73 m^−^^2^ in G5, almost all of the patients (96.7%) in the G5 group were on hemodialysis therapy because of kidney failure. On the other hand, very few patients (0.02%) were on hemodialysis in the G1‐4 group (Table 2). In the present study, we examined several clinical parameters, including the periodontal condition, and also performed analysis after classifying the patients into groups with or without DM. Recently, Grubbs reported a positive association between periodontal disease (classified as severe vs. non‐severe) and kidney dysfunction with or without DM in African American subjects (Grubbs et al., 2015). Prior investigation of Pima Indian adults with DM and an eGFR >60 ml min^−1^ 1.73 m^−2^ revealed that severe periodontal disease, diagnosed from the number of missing teeth and % alveolar bone loss on panoramic radiographs, was associated with a 3.5‐fold increase in the risk of kidney failure (Shultis et al., 2007). In older Japanese subjects, periodontitis has also been shown to become worse along with worsening eGFR category (classified as >60, 30–59, and <29 ml/min^−1^ 1.73 m^−2^) (Iwasaki et al., 2012). Furthermore, several studies have suggested a bidirectional relationship between glycemic control and periodontal disease (Okada et al., 1996; Nagasawa et al., 2010; Nishimura et al., 2003). It is generally accepted that elevated glucose levels promote the pathophysiology of periodontitis (Preshaw & Bissett, 2013). In the present study, we compared differences of the several periodontal parameters between patients with DM from the G1‐4 group and the G5 group. We demonstrated that the number of missing teeth, CPI, and OHI were all higher in the G5 with DM group than in the G1‐4 group among patients with DM (Figure 3). In general, alveolar bone loss by periodontitis may progress in short period of time, and tooth loss by periodontitis may occur in long period of time. Of course, dysfunction of kidney should be developed in long period of time by several causes. We think, therefore, that there was an association between kidney dysfunction and missing teeth, but not bone loss. Furthermore, poor oral hygiene may be reflected to the ability of self‐care in each patient. Probably, many DM patients in G5 stage may not able to maintain the oral hygiene themselves. OHI is a useful parameter for evaluation of oral hygiene, and poor oral hygiene must be a significant problem in DM patients with kidney dysfunction. Further study will be needed to clarify the clinical usefulness of oral care in the patients. Importantly, because we found that glycemic parameters of the G5 group were lower than those of the G1‐4 group (Table 2), kidney dysfunction has a strong influence on the periodontal conditions. Finally, to clarify the factors affected by kidney dysfunction, we focused on the G1‐4 group and statistically examined the relation between several parameters and eGFR. As shown in Figure 4, there was a correlation between eGFR and BP. In addition, there was also a correlation between eGFR and the number of missing teeth. Because no statistical relation was found between eGFR and age, the tooth loss could be a useful independent indicator of the impairment of kidney function.

There is growing clinical evidence to suggest a direct or indirect association between periodontitis and kidney dysfunction (Macisaac et al., 2014; Grubbs et al., 2015). Furthermore, there is a positive correlation between periodontal disease and obesity, hypertension, hyperlipidemia, or hyperglycemia (Zhou, Zhang, Liu, Zhang, & Li, 2015), although the molecular mechanisms of these relationships are unclear. Periodontitis is a low‐grade systemic inflammation that is reported to cause elevated systemic levels of inflammatory mediators such as C‐reactive protein and interleukin‐6 (Paraskevas, Huizinga, & Loos, 2008). A number of studies have found a similar association between an increase of C‐reactive protein or interleukin‐6 and increased mortality in dialysis patients (Zhang et al., 2013; Zimmermann et al., 1999). Unless oral infection is controlled, it is possible that the risk of kidney dysfunction associated with DM may be significantly increased due to periodontal infection. Circulating inflammatory cytokines due to periodontitis might be at least partly responsible for development of renal impairment, resulting in kidney failure.

This study had several limitations. First, it is not possible for a cross‐sectional study to obtain proof of a cause‐and‐effect relationship, so further prospective research will be required to confirm any causal relationship between kidney function and periodontal condition. Second, we classified the subjects by using eGFR. Recently, it has been reported that kidney function might be more accurately assessed by using both serum cystatin and creatinine levels in persons aged 70 years or older (Schaeffner et al., 2012). Thus, the appropriateness of our classification will need to be re‐evaluated in relation to geriatric medicine. Finally, we did not assess comorbidities besides DM, although other comorbidities may act as confounders and also affect periodontal condition. In the future, a risk‐adjusted approach could increase the validity of our findings.

## 5 CONCLUSIONS

A number of Japanese patients with advanced CKD have periodontal problems such as tooth loss that should be managed clinically near the start of dialysis. To confirm our findings, appropriate intervention trials will be needed to determine whether treating periodontitis reduces kidney dysfunction.

## CONFLICT OF INTEREST

The authors declare no conflicts of interest.
